# Paget’s Disease of Bone: A Rare Incidence in Early Adult Life in Pakistan, Southeast Asia

**DOI:** 10.7759/cureus.50242

**Published:** 2023-12-09

**Authors:** Ali H Rizvi, Hafsa Mobeen, Afaq Ahmad, Hina Saghir, Ahmed Farhan

**Affiliations:** 1 Internal Medicine, Shaheed Zulfiqar Ali Bhutto Medical University, Islamabad, PAK; 2 Internal Medicine, HBS Medical and Dental College, Islamabad, PAK; 3 General Surgery, Shaheed Zulfiqar Ali Bhutto Medical University, Islamabad, PAK; 4 Medicine, Shaheed Zulfiqar Ali Bhutto Medical University, Islamabad, PAK

**Keywords:** rare bone disease, case report, young adult, internal medicine and rheumatology, paget's disease of bone

## Abstract

Paget’s disease of bone (PDB) is a focal disorder of skeletal remodeling leading to alteration of bony structures and properties, uncommonly reported in people under the age of 40 years, particularly in the Southeast Asia region. In the literature, PDB has been well reported in regions of Europe, in contrast to Asia where there has been limited records of prevalence of PDM especially in the younger age population. Here, we report a 21-year-old male patient presenting with an advanced disease with complaints of auditory impairment, bone pain, and neurological signs due to compression of the interverbal disc developed progressively over two months, having an elevated serum alkaline phosphatase level of 601 IU/L. The patient was administered zoledronic acid that brought significant improvement in his health. We aim to signify the advanced presentation of PDB in our region to create awareness about the condition among physicians and aid in the prompt diagnosis and prevention of the most dreaded complications of the disease, such as osteosarcoma, secondary osteoarthritis, and fractures.

## Introduction

Paget’s disease of bone (PDB) is the dysfunction of the osteoclast-osteoblast cells leading to an abnormal bone turnover causing the formation of thick, brittle, and fragile bones. Due to the quick remodeling of the bones, their structures are altered and become prone to getting fractured, while often compressing the nerves passing near them. The most common complaints reported are hearing loss, bone pain, disfiguration of the craniofacial bones (lion-like face), and weakness in the extremities [1]. Generally, it affects the skull, axial skeleton, tibia, and fibula, and the patient can carry on with his/her life for a long time before noticing any symptoms. This disease was first reported by Sir James Paget in 1876 who called it "osteitis deformans." Even though it is widely reported in Europe, more specifically in the United Kingdom [2], it is considered extremely rare in Southeast Asia and mostly seen in people above 60 years of age in general. Limited literature is available in the region, regarding patients being under the age of 30 with three cases reported in India so far [3-4]. The etiology of this ailment is still unknown; however, certain genetic and environmental factors are suspected to have an influence. It also has an association with osteosarcoma, which often leads to severe bone pain [5]. The basic management involves administrating zoledronic acid [6] that slows the calcium loss from bones by reducing the osteoclast activity, hence stunting the further progression of the disease. Moreover, serum alkaline phosphatase level is recommended as a first-line screening test along with radionucleotide bone scans [7].

The case we present is of a young male from Pakistan that was diagnosed with PDB.

## Case presentation

**Figure 1:**
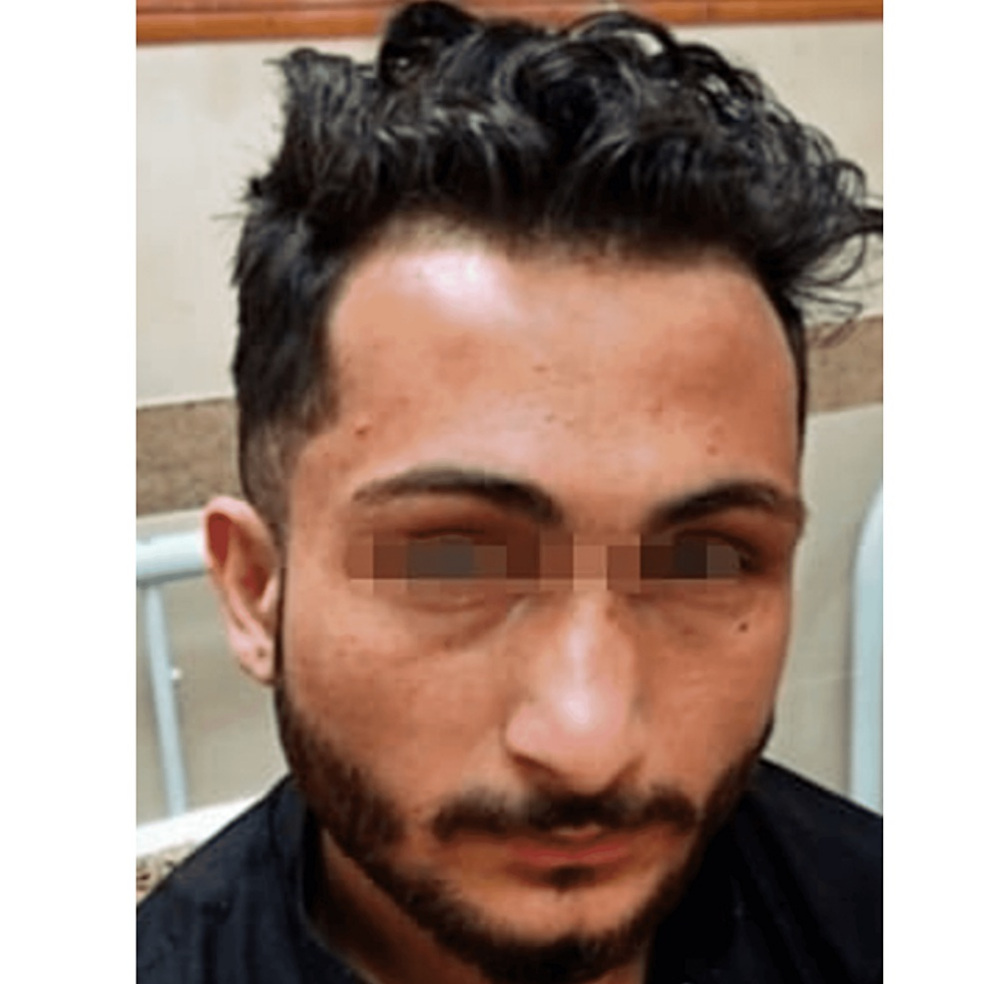
Patient image showing frontal bossing.

**Figure 2:**
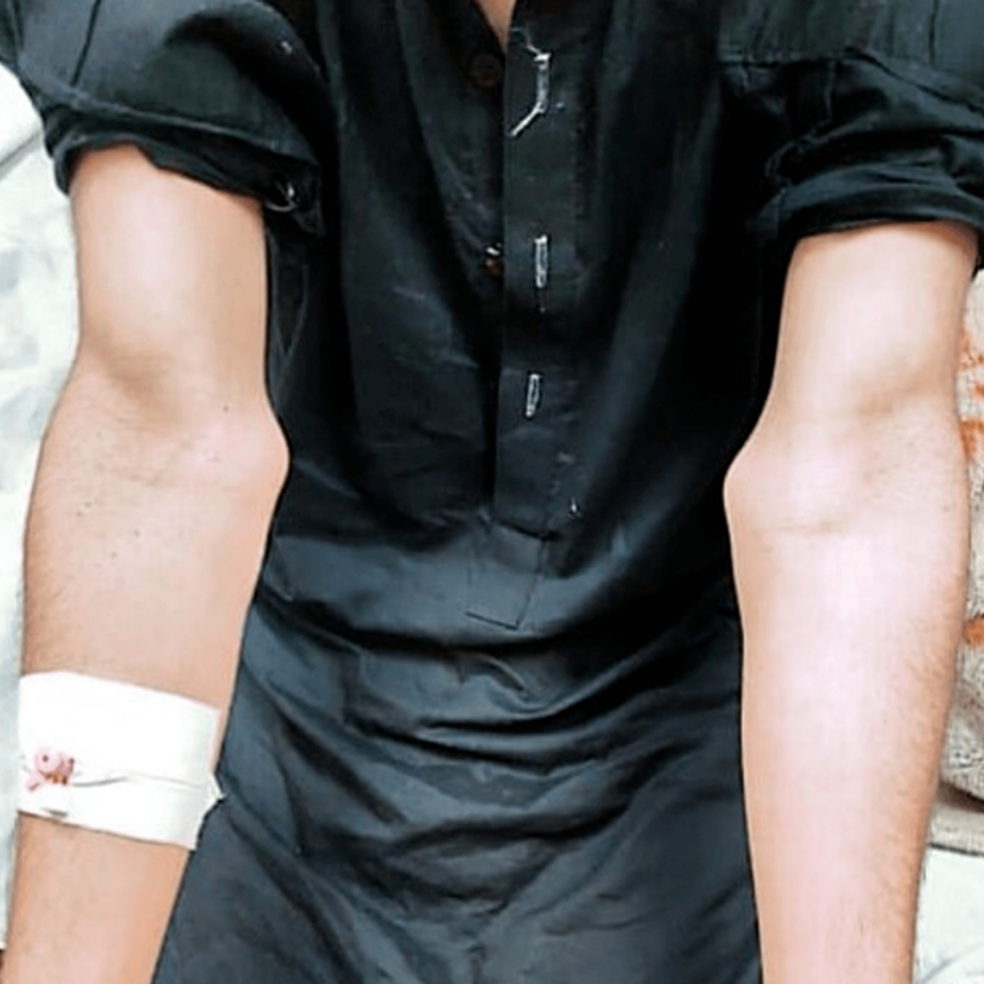
Patient image showing bony eminence at the elbow joints.

A 21-year-old male patient was referred from the neurology department to the internal medicine department with the suspicion of metastatic bone disease, at the Pakistan Institute of Medical Sciences (PIMS) Hospital, Islamabad, Pakistan, for his complaints of progressive backache and proximal muscle weakness for the past eight weeks. The patient had a history of difficulty in hearing since childhood along with generalized backache since eight months. His symptoms were worsening to an extent that he had difficulty in performing daily activities. Back pain was severely affecting his upper and lower back with associated shooting pains in the legs and arms. He had normal bowel and bladder control but had developed a stooped posture over time. There was no prior history of trauma, fever, weight loss, and night sweats or any history of corticosteroids intake. He had no history of joint pains, myalgia, difficulty in swallowing, or joint deformities. On examination, he had prominent frontal bossing (Figure 1), knocked knees, and bony prominence at elbows (Figure 2). He had generalized bony tenderness along with lower extremity weakness with a proximal muscle power of 3/5 and distal power of 4/5. He had bilateral lower extremity hyperreflexia with clonus.

Based upon his symptoms, the general physician and neurologist had carried extensive workup to rule out hyperparathyroidism, muscular dystrophy, metastatic bone disease, and hypothyroidism. Table 1 shows the results of the investigations done.

**Table 1:** Patient's lab investigation results.

Labs	Results	Units	Reference range
Serum calcium	10.4	mg/dL	8.4-10.4
Parathyroid hormone	30.2	pg/mL	8.7-79.6
Serum phosphorous	4.8	mg/dL	2.7-4.5
Alkaline phosphatase	601	U/L	<280
Vitamin D(25OH)	49.9	ng/mL	30-100
Serum testosterone	1091	ng/dL	300-1080
Thyroid-stimulating hormone (TSH)	3.0	mU/mL	0.4-5.5
C-reactive protein	0.5	mg/dL	0.8-1
Erythrocyte sedimentation rate (ESR)	4	mm/hr	0-15
Celiac serology	Negative	-	Negative
Lactate dehydrogenase(LDH)	349	U/L	140-280
Serum creatinine	0.74	mg/dL	0.7-1.2

**Figure 3:**
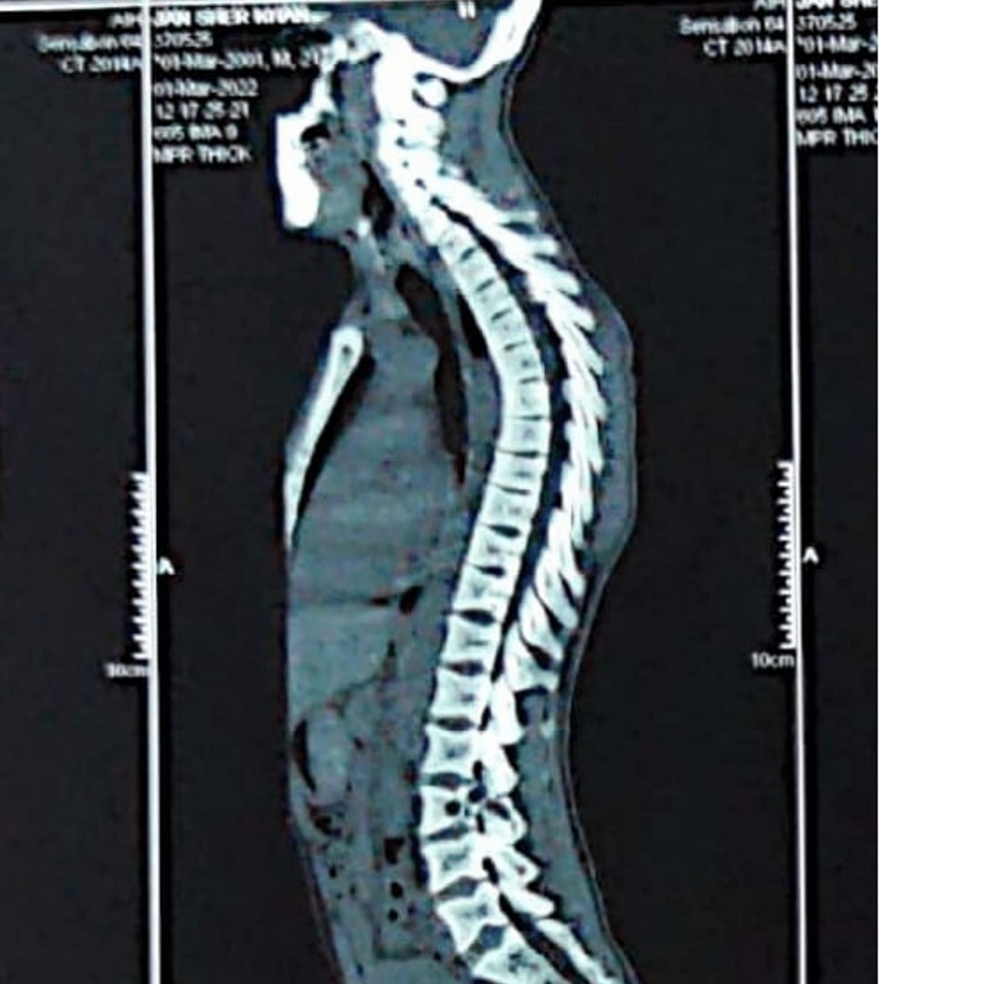
MRI spine without contrast suggestive of a complete collapse of the C3 vertebral body and partial collapse of the C4 vertebral body. There is accentuation of the lordotic curvature.

Muscle biopsy suggestive of mild degenerative and regenerative activity without fibrosin and inflammation ruled out muscular dystrophy. CT spine without contrast indicated diffuse osteopenia with cortical thinning and prominent trabecular pattern of all visualized bones with multilevel endplate erosive changes. There were multilevel lytic expansile lesions in the vertebral bodies and extending in the posterior element at the C3 and C4 levels causing canal stenosis at this level. This raised the suspicion of metastatic bone disease, which lead to further imaging assistance with magnetic resonance imaging (MRI) of the spine (Figure 3).

**Figure 4:**
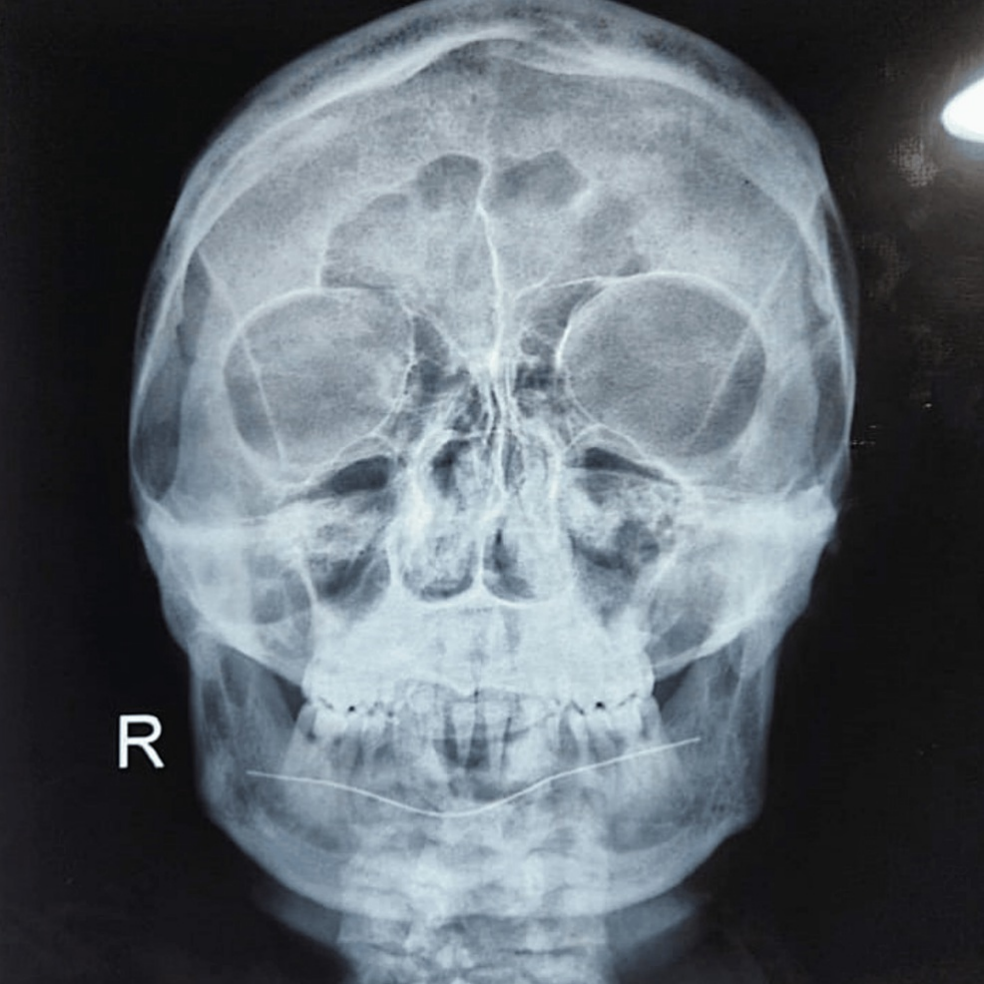
Skull X-ray showed bone expansion with widening of the calvarial diploic space and bone deformity. Alternating areas of osteolysis and osteosclerosis are suggestive of PDB.

**Figure 5:**
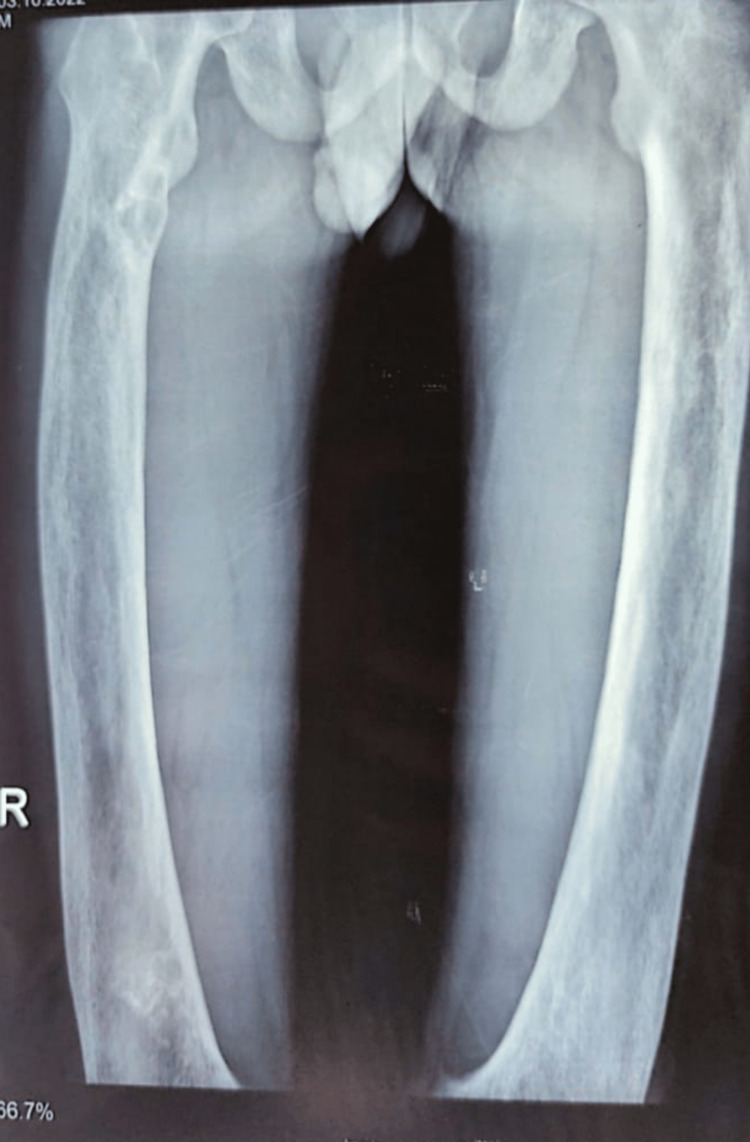
Radiograph of long bones showing cortical thickening and alternating osteolysis and osteosclerosis. There is lateral bowing of the femurs and a characteristic "blade of grass" sign present.

Considering the advanced presentation of the patient in a short period of time in such a young adult with associated compressive myelopathy made the clinician overlook the possibility of PDB, and hence the diagnosis became challenging. The characteristic radiological features of PDB present in this patient are illustrated in Figures 4-5. Individually, these features are not specific, but when they occur in combination, they are usually diagnostic.

An audiometry test showed mild conductive and sensorineural hearing loss in both his ears. Bone mineral density test indicated spine and distal forearm osteoporosis with hip joint osteopenia. A Z score of -2.9 in the lumber spine and forearm and a Z score of -1.9 in the hip joint were obtained.

**Figure 6:**
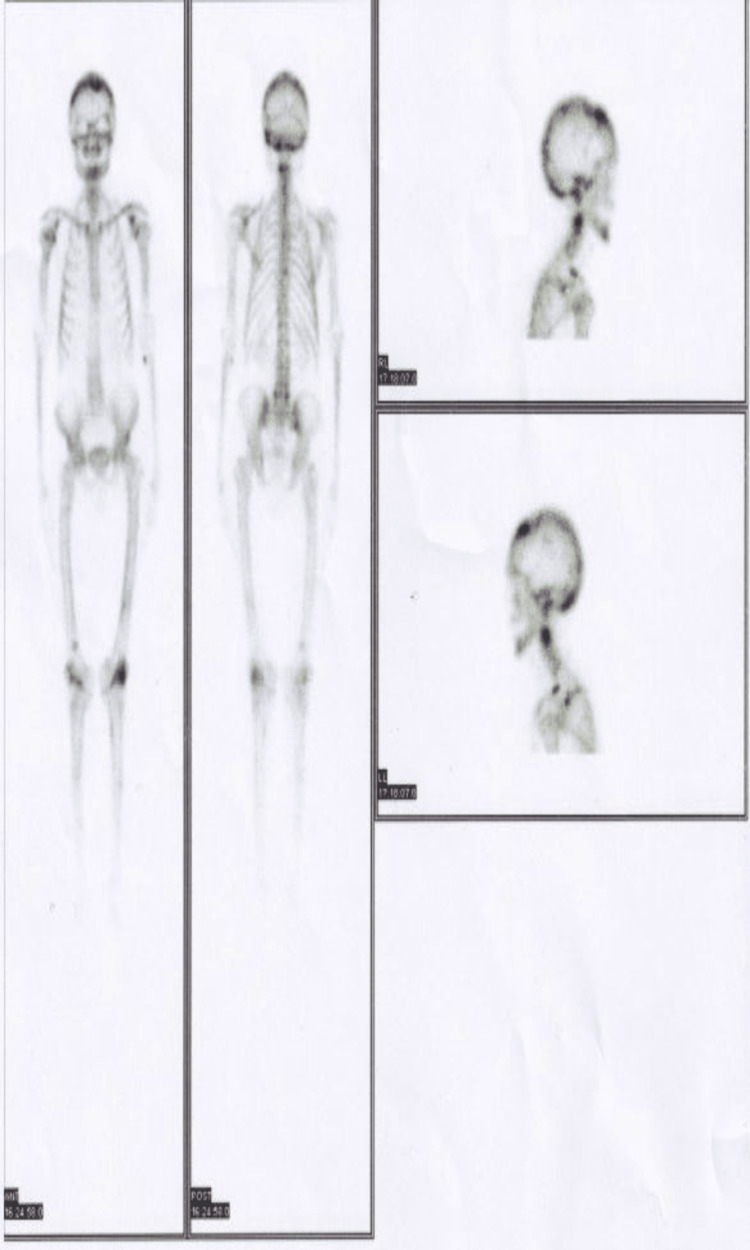
Technetium-99m whole body bone scan.

The technetium-99m whole body bone scan (Figure 6) showed an increased overall tracer uptake in the bones, such as the parietal region of the skull, maxillae, mandible, multiple cervical, thoracic and lumber vertebrae, bilateral ribs, bilateral femora and tibiae, humeri, radii, and pelvic bones.

The patient was administered with intravenous zoledronic acid 5 mg in a 500 ml normal saline solution with a single dose over a period of 15 minutes. The patient's response to treatment was remarkable, manifesting as a rapid return to ambulation with the assistance of aids within a few days. In addition, the patient demonstrated neurological improvement, exhibiting normal lower extremity power 5/5, reflexes, and tone. Notably, the patient's alkaline phosphatase (ALP) level decreased from 601 IU/L to 271 IU/L following the administration of zoledronic acid. Subsequently, the patient was discharged with a prescription for oral risedronate sodium 150 mg, to be taken once a month for the next two months, and was advised to follow up with the rheumatology department after six weeks’ time.

## Discussion

PDB is considered to be the second most common bone disorder after osteoporosis [8]. This condition manifests commonly in the elderly and rarely under the age of 35, with predilection in the males [9]. The deviation from the common distribution pattern of PDB leads to delayed diagnosis and advanced progression of the disease. 

While the condition is fairly well reported in European countries, it is often delayed in being diagnosed in Asian, African, and South American Countries mainly due to limited reports of such cases and lack of public health facilities available [10]. Similarly, the average delay in the diagnosis of a symptomatic patient in Tunisia has been reported to be three years [11].

Despite the typical presentation of the disease in our patient, diagnosis was challenging due to the paucity of literature evidence of the onset of PDB in the early twenties, with no family history and presenting progressive complaints in a short span of time. Only two case reports of PDB in Pakistan have been documented so far, with one being a 60-year-old female who was asymptomatic and came for preoperative evaluation for cholecystectomy and the other patient being a 70-year-old male who had complaints of difficulty in walking and pain in his right leg since five months [12-13]. Few cases have been documented globally involving individuals aged between 16 and 55 at the time of diagnosis, exhibiting a range of symptom durations spanning from three months to as long as 30 years before identification. These cases have been managed using a variety of approaches, including the administration of risedronate, pamidronate acid, calcitonin, and clodronate [14].

The patient experienced a swift progression of the disease, and the diagnosis was delayed primarily due to an uncommon presentation and, secondarily, the lack of diagnostic radiological facilities in remote areas of the country. ALP levels were significantly elevated, a key diagnostic marker for this condition [15]. Based on radiological findings, a provisional diagnosis was initially established. A technetium-99m whole body bone scan revealed involvement in multiple bones, while MRI and CT scans indicated lesions in the skull, vertebrae, and long bones. The patient received intravenous zoledronic acid 5 mg [16-17], leading to a notable improvement in mobility within a few days. Regular follow-ups were scheduled to monitor for osteosarcoma and assess any changes in hearing.

## Conclusions

Despite the uncommon occurrence of this condition in individuals of young age, our patient serves as a significant example for physicians to remain vigilant regarding such cases in Pakistan. The case report emphasizes the typical symptoms and diagnostic procedures associated with PDB. Enhanced awareness and attentiveness are crucial at present to ensure prompt diagnosis and effective management of such cases, potentially averting severe complications similar to those experienced by our patient.
